# Supplementary material for: Urinary complement proteins in IgA nephropathy progression from a relative quantitative proteomic analysis
Source: PeerJ. 2023 Apr 11;11:e15125. doi: 10.7717/peerj.15125 (PMC10103701; doi:10.7717/peerj.15125)
Supplement: Supplemental Information 2 [file peerj-11-15125-s002.doc]

**Supplemental Table 2. Differential complement proteins between** **glomeruli and urine of IgAN patients**

| Glomeruli in IgAN(18 complement proteins) | Urine in IgAN (27 complement proteins) |
| --- | --- |
| C3 | C3 |
| C1s | C1s |
| C1r | C1r |
| C1q | C1q |
| SERPING1 | SERPING1 |
| C2 | C2 |
| C4 | C4 |
| CFB | CFB |
| CFH | CFH |
| CFHR2 | CFHR2 |
| CFHR4 | CFHR4 |
| C5 | C5 |
| C6 | C6 |
| C7 | C7 |
| C8A | C8A |
| C8B | C8B |
| C8G | C8G |
| C9 | C9 |
| nd | MCP |
| nd | MBL2 |
| nd | Ficolin-2 |
| nd | MASP1 |
| nd | MASP2 |
| nd | CFD |
| nd | Properdin |
| nd | DAF |
| nd | CFI |

Abbreviation: nd: not detectd. The red fonts represent the differential complement proteins of LP and AP between glomeruli and urine of IgAN patients. The black fonts refer to the common complement proteins.

**Reference**

1. Paunas TIF, Finne K, Leh S, Marti HP, Mollnes TE, Berven F, Vikse BE. Glomerular abundance of complement proteins characterized by proteomic analysis of laser-captured microdissected glomeruli associates with progressive disease in IgA nephropathy. Clin Proteomics. 2017 Aug 14;14:30. doi: 10.1186/s12014-017-9165-x. PMID: 28814945.
