# Supplementary figures and images for: Urinary complement proteins in IgA nephropathy progression from a relative quantitative proteomic analysis
Source: PeerJ. 2023 Apr 11;11:e15125. doi: 10.7717/peerj.15125 (PMC10103701; doi:10.7717/peerj.15125)

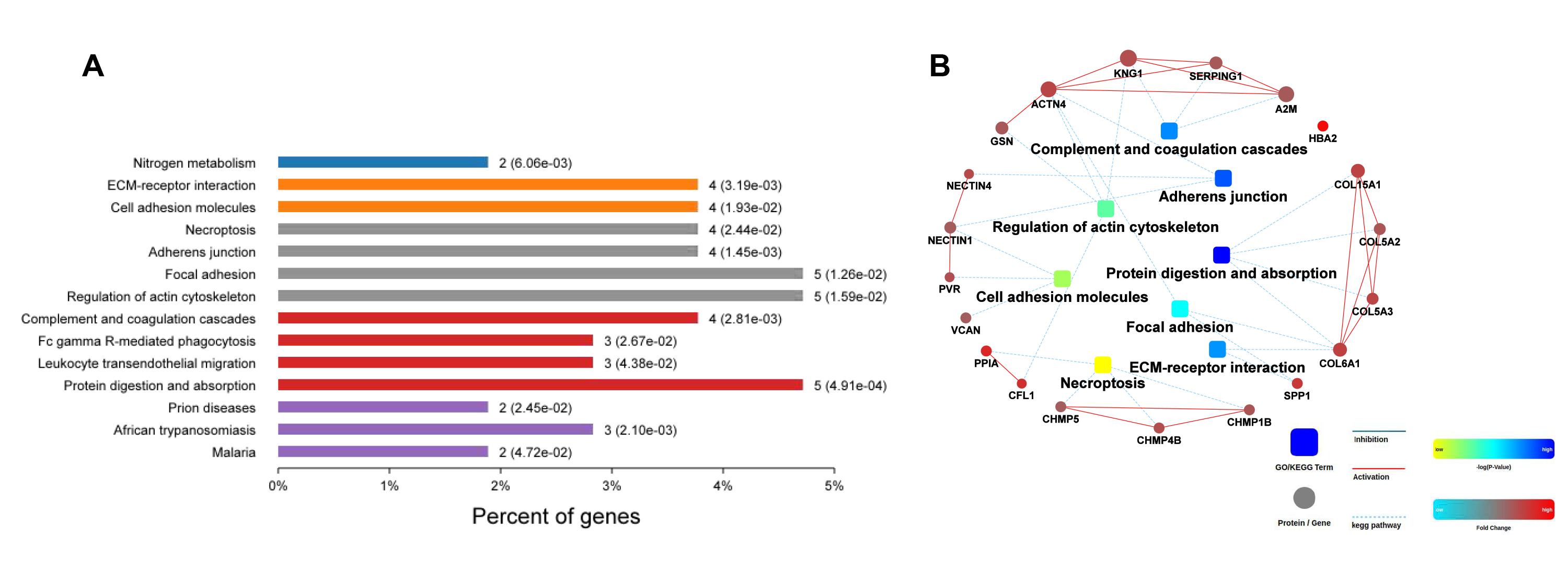

Supplement: Supplemental Information 3 — (A) KEGG pathway enrichment analysis of 107 upregulated proteins in IgAN-1 compared to pMN. (B)The PPI networks were created for 107 upregulated proteins in IgAN-1 compared to pMN. [file peerj-11-15125-s003.png]

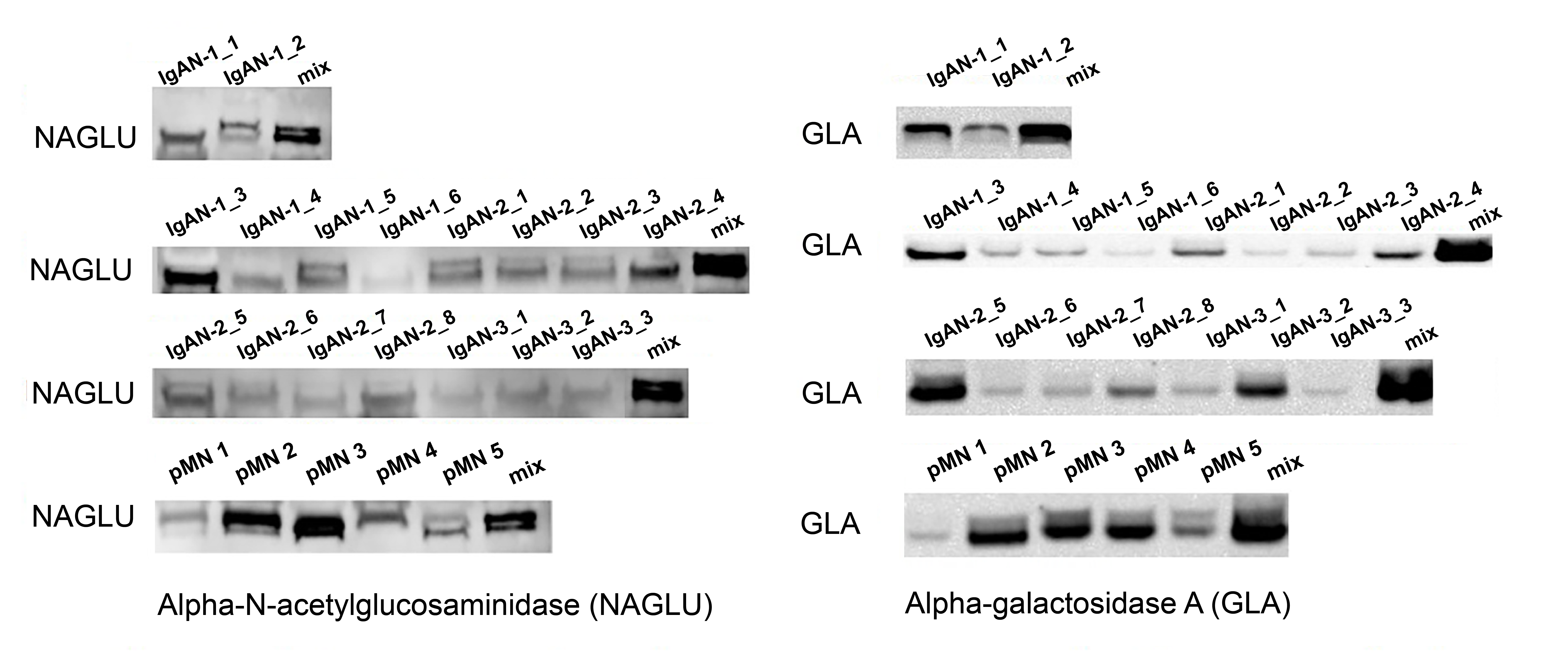

Supplement: Supplemental Information 4 [file peerj-11-15125-s004.png]

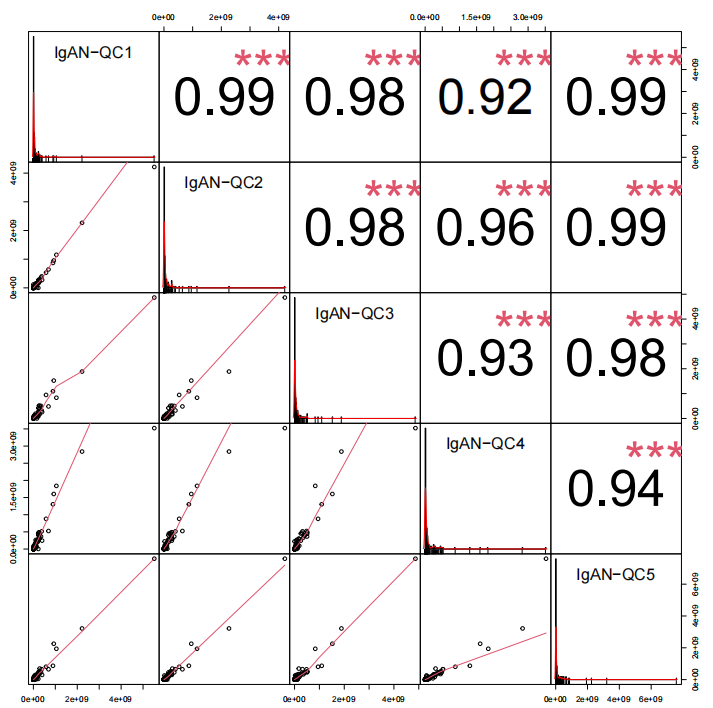

Supplement: Supplemental Information 5 [file peerj-11-15125-s005.png]

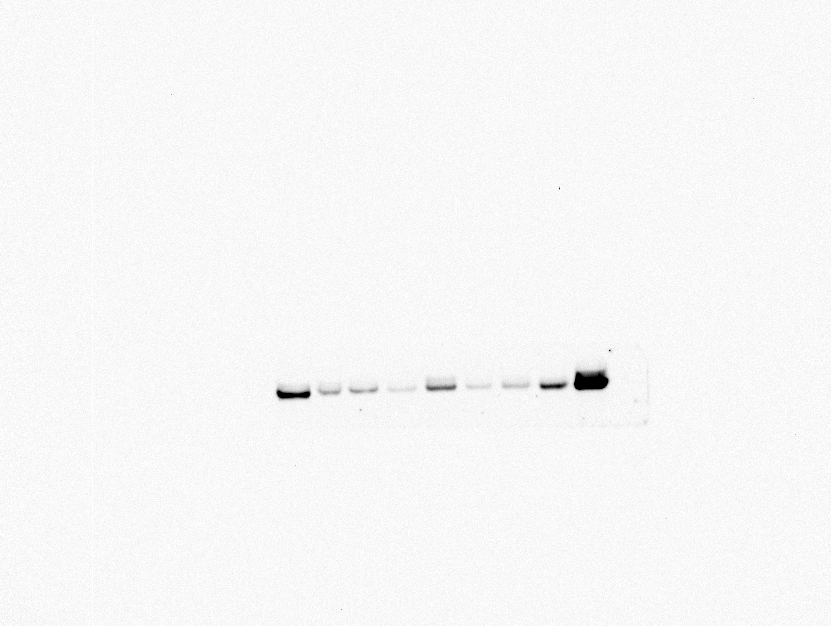

Supplement: Supplemental Information 7 [file peerj-11-15125-s007.zip › GLA 1.png]

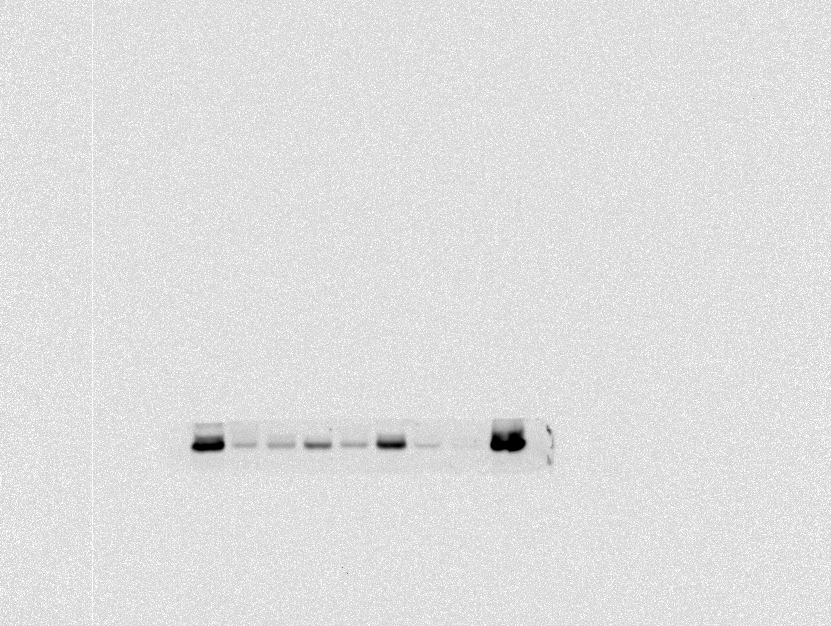

Supplement: Supplemental Information 7 [file peerj-11-15125-s007.zip › GLA 2.png]

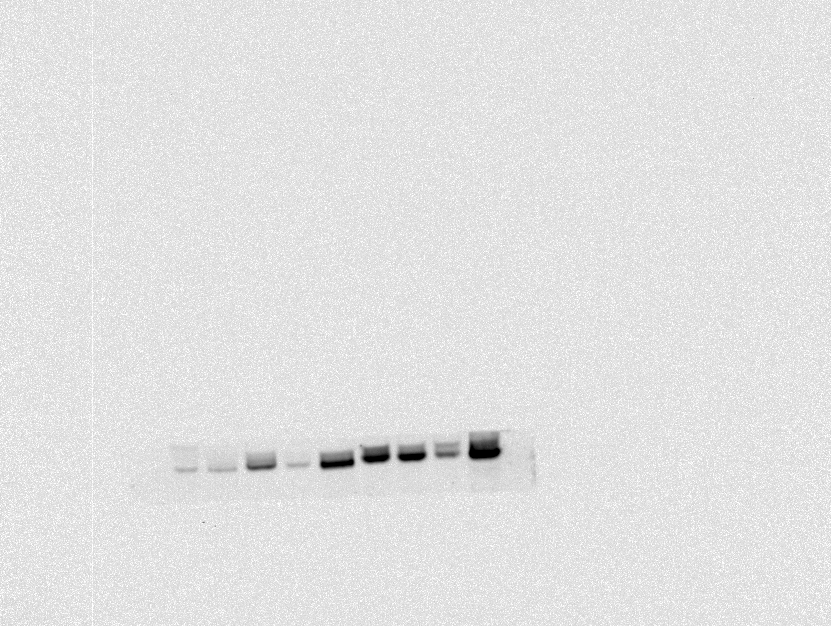

Supplement: Supplemental Information 7 [file peerj-11-15125-s007.zip › GLA 3.png]

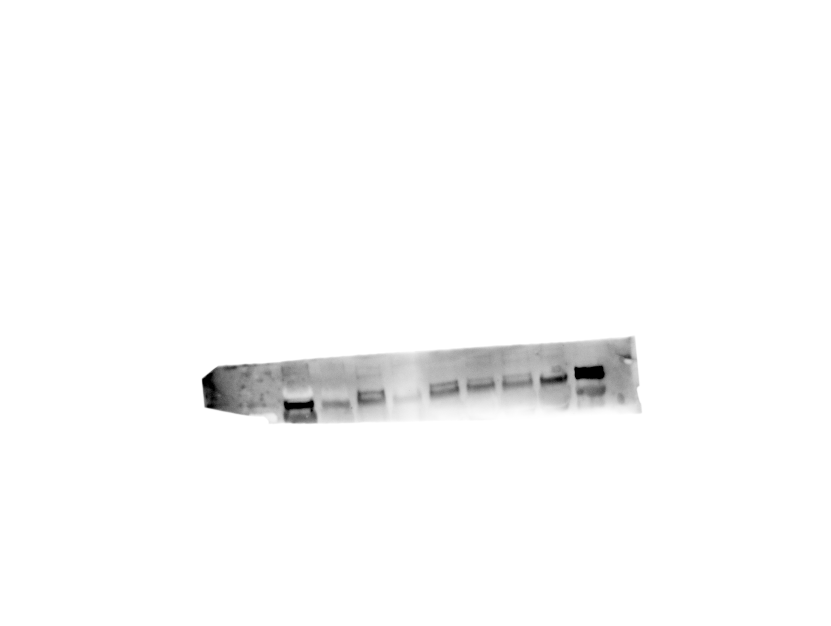

Supplement: Supplemental Information 7 [file peerj-11-15125-s007.zip › NAGLU 1.png]

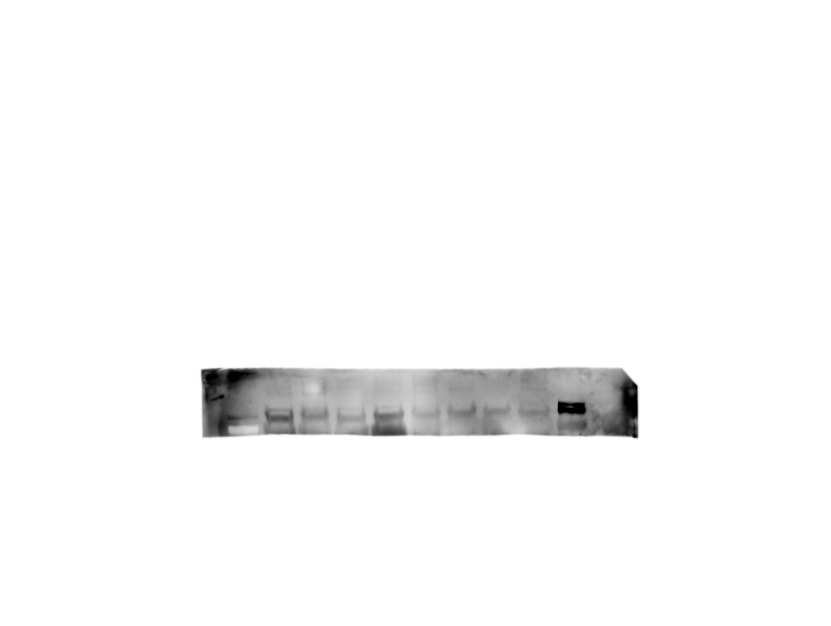

Supplement: Supplemental Information 7 [file peerj-11-15125-s007.zip › NAGLU 2.png]

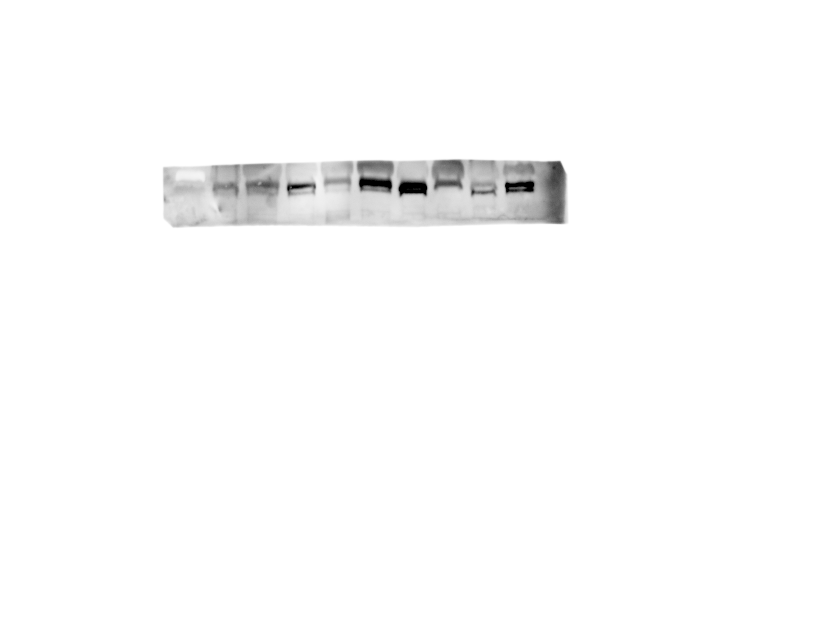

Supplement: Supplemental Information 7 [file peerj-11-15125-s007.zip › NAGLU 3.png]
