# Supplementary material for: Urinary complement proteins in IgA nephropathy progression from a relative quantitative proteomic analysis
Source: PeerJ. 2023 Apr 11;11:e15125. doi: 10.7717/peerj.15125 (PMC10103701; doi:10.7717/peerj.15125)
Supplement: Supplemental Information 6 [file peerj-11-15125-s006.docx]

The raw data for LC-MS/MS analysis can be download from Figshare, including iTRAQ and PRM.

iTRAQ-IgAN: 10.6084/m9.figshare.21685898

PRM-1-IgAN: 10.6084/m9.figshare.21687299

PRM-2-IgAN: 10.6084/m9.figshare.21688946

PRM-3-IgAN: 10.6084/m9.figshare.21688979

PRM-4-IgAN: 10.6084/m9.figshare.21689000
